# Resilience Differences Among Health Professionals: Examining the Impact of Body Image Appreciation

**DOI:** 10.7759/cureus.80745

**Published:** 2025-03-17

**Authors:** Georgios Manomenidis, Dimitrios Kosmidis, Maria Lavdaniti, Anna Tsiakiri, Maria Amanatidou, Ioannis Koutelekos, Sotiria Loukidou, Vaios Kalatzis, Vasiliki Georgousopoulou

**Affiliations:** 1 Department of Nursing, Democritus University of Thrace, Alexandroupolis, GRC; 2 Department of Nursing, International Hellenic University, Thessaloniki, GRC; 3 Medical School, Democritus University of Thrace, Alexandroupolis, GRC; 4 Department of Nursing, University of West Attica, Athens, GRC; 5 Department of Nursing, General Hospital of Ptolemaida, Ptolemaida, GRC; 6 Department of Surgical Anatomy, General Hospital of Ptolemaida, Ptolemaida, GRC

**Keywords:** body image appreciation, health professionals, nurses, physicians, resilience

## Abstract

Introduction: Body image (BI) is a determinant of mental health and has a strong association with self-esteem and self-worth, core elements of resilience. The relationship between BI appreciation and resilience in health professionals (HPs) remains largely unexplored.

Aim: The aim of the study was to compare HPs’ resilience and determine the relationship of resilience with BI appreciation.

Methods: A cross-sectional comparative study was conducted using an online questionnaire that was completed by 484 HPs that included a) demographic and occupational characteristics, b) the Body Appreciation Scale-2 (BAS-2), and c) the Connor-Davidson Resilience Scale (CD-RISC-10). Data was collected between January and March 2024.

Results: Nurses reported significantly higher BI (3.96 ± 0.60 vs. 3.45 ± 0.73, p < 0.001) and resilience (3.25 ± 0.64 vs. 2.98 ± 0.55, p < 0.001) compared to physicians. Multiple regression analysis identified BI as a strong positive predictor of resilience for both nurses and physicians (p < 0.001). Nurses’ resilience was also positively associated with increased working experience (p = 0.001) and negatively with the managerial position (p < 0.001) and higher level of education (p = 0.003). Physicians’ resilience was also positively linked to cohabitation (p = 0.001) and the existence of children (p = 0.006) while negatively associated with working in internal departments (p = 0.001).

Conclusions: BI appreciation appears to play a significant role in HPs’ resilience, while professional and personal factors influence each group differently. Interventions targeting in enhancing BI appreciation among HPs are suggested.

## Introduction

In our fast-paced world, time is considered valuable, and people tend to evaluate and judge others' personalities by focusing mainly on their appearance, either by positive or negative appraisals [1]. Individuals are constantly bombarded by information and images from social media, in which appearance should match the societal proposed standards while certain body types and ideals are promoted, with a plausible harmful influence on body image (BI) and mental health [2].

BI is a complex construct consisting of individuals' perceptions, thoughts, feelings, behaviors, and attitudes regarding the body [3]. Age, gender, family, peers, public opinion, and ethnicity are some of the elements that affect the way people perceive their BI [4]. According to Thomas F. Cash’s definition, BI is “a multidimensional construct encompassing self-perceptions and attitudes regarding one’s physical appearance” [5]. 

Resilience is a critical psychological trait that enables individuals to effectively cope with stress, adversity, and challenges. It is closely associated with mental well-being and adaptability in stressful conditions, serving as a key protective factor against burnout and psychological trauma [6,7]. For healthcare professionals working in high-pressure environments, resilience is particularly vital as it influences both their personal well-being and professional performance. Concurrently, BI perception has emerged as a significant determinant of mental health, with positive BI linked to higher self-esteem, life satisfaction, and lower levels of anxiety and depression [8]. Furthermore, Avalos and Tylka [9] argued that positive BI contributes to building a stable internal support system, enhancing resilience against challenges. Lokyan et al. [10] highlighted the role of psychological training in enhancing emotional intelligence and resilience, particularly in high-stress environments. Their findings emphasize the importance of structured training in managing stress and promoting personal development. Recent research on healthcare professionals, including students, has further reinforced these associations. For instance, Kumar et al. [11] highlighted the role of positive BI in fostering emotional resilience among medical students, underlining its impact on mental well-being and stress management. Similarly, Chen et al. [12] highlighted the role of mindfulness in fostering resilience, with perceived stress acting as a mediator. Their findings reinforce the importance of internal psychological resources in maintaining well-being and adaptability among healthcare professionals.

Recent research has increasingly focused on the interplay between resilience and BI. Meland et al. [13] demonstrated that individuals with positive BI exhibited greater self-esteem and self-confidence, which are critical for managing challenging circumstances. According to Tomlinson et al. [14], dispositional mindfulness is positively associated with psychological health, contributing to reduced psychopathological symptoms and improved emotional regulation. Combined with positive BI, mindfulness enhances psychological resilience, helping individuals to stay present and manage stress effectively. This link highlights the role of self-acceptance and emotional regulation in enhancing resilience, particularly in the context of healthcare professionals who are exposed to increased psychological challenges. Matera et al. [15] found that positive BI was directly associated with psychological well-being, with BI coping strategies playing a mediating role in reducing stress and enhancing adaptation to challenging situations. Similarly, Grogan [16] emphasized the protective role of positive BI in reducing stress and burnout, particularly among healthcare professionals. McCann et al. [17] also highlighted the role of resilience in mitigating burnout among nurses, underscoring the value of educational and psychological interventions in strengthening this trait.

While existing literature underscores the relationship between BI and resilience, limited research has focused on how these elements differently interact among the two largest healthcare professionals, namely nurses and physicians. Given the unique demands and stressors these groups face, understanding the connection between BI perception and resilience is crucial for developing targeted interventions to promote their mental well-being and professional performance.

The aim of the study was to address this gap by evaluating and comparing nurses’ and physicians’ BI perceptions and by examining the relationship between BI and resilience. By exploring these dimensions, the study seeks to contribute to the broader understanding of how psychological and personal factors influence the adaptability and mental health of these two groups in the healthcare setting.

## Materials and methods

A convenience sample of health professionals (HPs) participated in this online cross-sectional study between January 1st and March 30th, 2024. Participants were recruited through online forums for nurses and physicians, as well as social networking sites. Researchers posted an invitation to the study and requested individuals to participate in the online survey. Those who agreed followed a Web URL link to the questionnaire, hosted on Google Forms. The inclusion criteria were (1) being a nurse or a physician, (2) having permanent employment status, and (3) having at least one year of hospital work experience. The exclusion criteria included temporary employment status or less than one year of professional experience. The questionnaire took approximately 10-15 minutes to complete.

Measures

A cross-sectional comparative study was conducted using an online questionnaire completed by 484 HPs, which included demographic and occupational characteristics, the Body Appreciation Scale-2 (BAS-2), and the Connor-Davidson Resilience Scale (CD-RISC-10).

Demographic and professional characteristics

The demographic and occupational characteristics questionnaire was constructed for the purpose of the study and included questions about gender, age, parental status, and living arrangement as well as information about occupational characteristics (e.g. years of experience, managerial position, working department).

The Body Appreciation Scale (BAS-2)

BAS-2 [8] is a 10-item five-point Likert scale with responses ranging from 1 (never) to 5 (always). Higher scores indicate higher body appreciation. To calculate one’s final body appreciation score, item responses are summed, resulting in a score between 10 and 50. The BAS-2 scale has valid psychometric properties and has been translated and validated in the Greek population [18]. Cronbach’s alpha value for the study was 0.924, indicating excellent internal consistency.

The 10-item Connor-Davidson Resilience Scale (CD-RISC-10)

CD-RISC-10 [6] consists of 10 items describing different aspects of resilience that include flexibility, self-efficacy, emotion regulation, optimism, and cognitive focus/maintaining attention under stress. Each item is assigned to a five-item Likert scale ranging from 0 (not true) to 4 (true nearly all time). Overall score can range from 0 to 40, with higher scores indicating greater resilience. The CD-RISC-10 has been translated and validated in the Greek population [19]. The Cronbach’s alpha for the study was 0.888, indicating good reliability.

Ethical considerations

To address ethical concerns arising from the research procedure, a cover letter accompanied the questionnaire. Participants were informed of their right to withdraw from the study at any time. The study was conducted in adherence to the Declaration of Helsinki [20].

Statistical analysis

Data were entered into SPSS software (version 22.0 for Windows, IBM Corp, Armonk, NY). Categorical variables were presented as percentages while continuous variables were presented as means (SD=standard deviation). The Kolmogorov-Smirnov test was used to test the normality of the distribution. Since the regularity check showed no normal distribution of variables, nonparametric methods were conducted. A nonparametric Mann-Whitney U test was conducted to examine the differences in perceptions related to the variables of BAS-2. A multivariate linear regression analysis was performed to investigate the effect of various predictors on the variation in nurse’s and physicians’ resilience. The stepwise regression analysis was utilized. Stepwise selection was based on criteria of entry (p ≤ 0.05) and removal (p ≥ 0.10), ensuring the inclusion of variables with the highest statistical relevance.

## Results

A total of 484 questionnaires were received. Nurses made up 60.3% of participants, while physicians accounted for 39.7%. The average participant age was 41.34 years (SD = 10.24), with a mean of 15.2 years of work experience (SD = 9.52). Most participants were female (73.8%), cohabiting (75.2%), and working in internal departments (55.8%). Table 1 presents the demographic and professional characteristics.

**Table 1:** Demographic/professional characteristics of nurses and physicians (n = 484) This table presents demographic and professional characteristics of a sample of nurses (n = 292, 60.3%) and physicians (n = 192, 39.7%), along with p-values to indicate statistical differences between the two groups. p < 0.001 for statistically significant comparisons. *Values indicate mean ± SD. ICU, Intensive care unit; OR, operation room.

Demographic/professional characteristics	Total, n (%)	Nurses, n (%); N = 292 (60.3%)	Physicians, n (%); N = 192 (39.7%)	p-Value
Gender				<0.001
Male	127 (26.2)	50 (17.1)	77 (40.1)	
Female	357 (73.8)	242 (82.9)	115 (59.9)	
Age*	41.3 (±10.2)	43.47 (±9.4)	38.09 (±10.6)	<0.001
Living arrangement				<0.001
Alone	120 (24.8)	53 (18.2)	67 (34.9)	
Cohabitation	364 (75.2)	239 (81.8)	125 (65.1)	
Children				<0.001
Yes	282 (58.3)	203 (69.5)	79 (41.1)	
No	202 (41.7)	89 (30.5)	113 (58.9)	
Working department				0.561
Internal department	270 (55.8)	166 (56.8)	104 (54.2)	
ICU/OR	214 (44.2)	126 (43.2)	88 (45.8)	
Working experience*	15.2 (±9.5)	18.3 (±8.8)	10.5 (±8.6)	<0.001
Education level				
No	177 (36.6)	101 (34.6)	76 (39.6)	0.265
Yes	307 (63.4)	191 (65.4)	116 (60.4)	
Managerial position				<0.001
Yes	267 (55.2)	144 (49.3)	123 (64.1)	
No	217 (44.8)	148 (50.7)	69 (35.9)	

Nurses scored significantly higher on all items of BAS-2, with an overall mean score of 3.96 (±0.60) compared to 3.45 (±0.733) of physicians (p < 0.001), indicating a higher level of body appreciation (Table 2). Appendix Table 6 is the Body Appreciation Scale questionnaire.

**Table 2:** Descriptive characteristics of BAS-2 among nurses and physicians This table presents the BAS-2 scores for nurses and physicians, measuring their attitudes toward body appreciation. *p < 0.05 for statistically significant comparisons. All the p-values being <0.001 indicate that all comparisons between the two groups are statistically significant. BAS-2, Body Appreciation Scale-2.

Body Appreciation Scale (BAS-2)	Nurses	Physicians	p-Value*
Variables	Mean (±SD)	Mean (±SD)	
1. I respect my body.	4.26 (±0.76)	3.78 (±0.85)	<0.001
2. I feel good about my body.	3.74 (±0.85)	3.36 (±0.82)	<0.001
3. I feel that my body has at least some good qualities.	4.11 (±0.67)	3.8 (±0.88)	<0.001
4. I take a positive attitude toward my body.	3.8 (±0.87)	3.4 (±0.97)	<0.001
5. I am attentive to my body’s needs.	3.57 (±0.85)	3.12 (±1.02)	<0.001
6. I feel love for my body.	4.02 (±0.78)	3.52 (±1.04)	<0.001
7. I appreciate the different and unique characteristics of my body.	4 (±0.87)	3.46 (±0.96)	<0.001
8. My behavior reveals my positive attitude toward my body; for example, I hold my head high and smile.	4.18 (±0.88)	3.45 (±0.97)	<0.001
9. I am comfortable with my body.	3.91 (±0.86)	3.31 (±1.1)	<0.001
10. I feel like I am beautiful even if I am different from media images of attractive people (e.g., models, actresses/actors).	3.99 (±0.76)	3.33 (±0.97)	<0.001
Total score of BAS	3.96 (±0.60)	3.45(±0.733)	<0.001

Similarly, regarding resilience, nurses outperformed physicians with an overall mean score of 3.25 (±0.643) compared to 2.98 (±0.552) (p < 0.001) indicating higher resilience (Table 3). Appendix Table 7 is the Resilience questionnaire (CD-RISC-10).

**Table 3:** Descriptive characteristics of resilience among nurses and physicians This table presents the resilience levels measured by the CD-RISC-10 in nurses and physicians, assessing their ability to adapt to stress, recover from adversity, and maintain emotional strength. *p < 0.05 for statistically significant comparisons. CD-RISC-10, Connor-Davidson Resilience Scale.

Resilience (CD-RISC-10)	Nurses	Physicians	p-Value*
Variables	Mean (±SD)	Mean (±SD)	
Adapt to change	3.55 (±0.75)	3.27 (±0.8)	<0.001
Deal with whatever comes my way	3.37 (±0.8)	3.01 (±0.78)	<0.001
See the humorous side of things	2.93 (±1.05)	2.85 (±0.98)	0.225
Stress makes me stronger	3.15 (±1.02)	2.84 (±0.94)	<0.001
Bounce back after illness or injury	3.25 (±1)	3.18 (±0.93)	0.184
Believe I can achieve goals despite obstacles	3.45 (±0.78)	3.22 (±0.7)	<0.001
Under pressure, I stay focused	3.26 (±0.84)	3.12 (±0.69)	0.004
Not easily discouraged by failure	2.85 (±0.9)	2.28 (±1.2)	<0.001
Think of myself as a strong person when facing challenges	3.48 (±0.64)	3.09 (±0.88)	<0.001
Able to handle unpleasant feelings	3.22 (±0.88)	2.86 (±0.89)	<0.001
Total score of resilience	3.25 (±0.643)	2.98 (±0.552)	<0.001

Relationship between dependent variable resilience and independent variables in nurses

Multivariate linear regression analyses revealed that higher BI appreciation and longer working experience were the strongest positive predictors of resilience, while the managerial position and a higher level of education negatively affected resilience. Twenty-one percent of the variance of resilience was explained by the above four independent variables; see Table 4.

**Table 4:** Multivariate regression analysis of predictors of resilience among nurses This table presents the results of a multiple regression analysis, showing the effect of four independent variables (BAS-2, managerial position, working experience, and education level) on an unspecified dependent variable (likely resilience, psychological well-being, or another outcome of interest). R² = 21%, p-value for ANOVA <0.001. BAS-2, Body Appreciation Scale-2; ANOVA, analysis of variance.

Independent variable	Unstandardized coefficient (b)	95% CI for b	p-Value
BAS-2	0.331	[0.215, 0.446]	<0.001
Managerial position	-0.251	[-0.388, -0.113]	<0.001
Working experience	0.014	[0.005, 0.022]	0.001
Education level	-0.242	[-0.402, -0.083]	0.003

Relationship between dependent variable resilience and independent variables in physicians

A similar regression analysis with the same variables was also performed to examine predictors of resilience among physicians. Higher BI appreciation, cohabitation, having children, having longer working experience, and working in an internal department were significantly associated with higher resilience. Thirty-six percent of the variance of resilience was explained by these six independent variables; see Table 5.

**Table 5:** Multivariate regression analysis of predictors of resilience among physicians This table presents multiple regression analysis results, indicating how different independent variables (e.g., BAS-2, living arrangement, children, working department, and working experience) predict an unspecified dependent variable (possibly resilience, psychological well-being, or another work-related factor). R² = 36%, p-value for ANOVA <0.001. BAS-2, Body Appreciation Scale-2; ANOVA, analysis of variance.

Independent variable	Unstandardized coefficient (b)	95% CI for b	p-Value
BAS-2	0.197	[0.096, 0.297]	<0.001
Living arrangement	0.185	[0.085, 0.285]	0.001
Children	0.226	[0.067, 0.384]	0.006
Working department	-0.268	[-0.414, -0.122]	0.001
Working experience	0.035	[0.015, 0.055]	0.001

## Discussion

The findings of this study highlight significant differences in perceptions and psychological resilience between nurses and physicians, offering valuable insights into the factors influencing their overall well-being and professional performance. Specifically, nurses demonstrated a more positive BI and a higher body appreciation score compared to physicians, reflecting greater acceptance and positivity toward their physical appearance. Nurses have probably realized the indirect association between positive BI and improved outcomes in well-being; thus, they actively pursue their resilience through self-care habits that optimize their BI, namely through exercise and healthy eating [21]. Caring for their physical appearance and BI is a clear indication of their efforts to increase their self-esteem and worth. By engaging in self-care practices, nurses mitigate stress and exhaustion related to the field of nursing practice [22]. On the other hand, physicians, due to their heightened responsibilities and critical decision-making roles, prioritize their patients’ needs at the expense of their own, by neglecting to implement self-care practices, including those referring to their BI improvement. 

In terms of resilience, nurses scored higher on aspects such as adaptability, the ability to stay focused under pressure, and less discouragement by failure. These differences suggest that nurses possess stronger mechanisms for adaptation and well-being under adverse conditions, likely due to the nature of their work, which often involves problem-solving and teamwork in challenging situations. McCann et al. [17] highlighted that teamwork and emotional support bolster confidence in one’s ability to achieve goals and manage challenges effectively. The lower resilience observed in physicians is possibly linked to their increased professional responsibilities and expectations for flawless performance. Shanafelt et al. [23] noted that the pressures in leadership and decision-making roles can diminish psychological flexibility and increase the risk of professional burnout. Physicians may also perceive failure as more significant and threatening, negatively impacting their ability to remain focused under pressure. In contrast, nurses appear to view failure as part of a continuous learning and improvement process, enhancing their emotional intelligence and resilience, a concept that aligns with modern approaches to psychological training in high-stress professions, as discussed by Lokyan et al. [10].

Although nurses scored higher on most resilience variables, the absence of differences in aspects such as "seeing the humorous side of things" and "bouncing back after illness or injury" suggests that these resilience dimensions are universal and not role-dependent. Connor and Davidson [6] noted that humor and recovery from illness are universal facets of psychological resilience. Work experience also appeared to enhance resilience, indicating that professionals with greater experience possess more developed skills for adaptation and stress management. Wasylkiw et al. [24] underscored that accumulated experience facilitates effective handling of challenges and boosts confidence. However, the negative association between educational level and resilience suggests that higher levels of education may be accompanied by increased demands and pressures [10,22].

The multivariate regression analysis identified BI appreciation and working experience as positive predictors of resilience among nurses. These findings suggest that nurses with a positive BI and extensive professional experience are more resilient, likely due to their enhanced self-esteem, adaptability, and confidence in their abilities to handle workplace stress. This is consistent with other studies conducted among HPs that identified high levels of self-esteem and increased working experience as protective factors of resilience [25]. Conversely, having a managerial position and higher educational levels negatively influenced resilience. This negative association might be attributed to the added pressures and expectations that come with advanced roles and qualifications [22].

BI appreciation also emerged as a significant positive predictor for physicians’ resilience, by reinforcing the role of self-esteem and self-perception in stress management. Additional positive predictors included cohabitation, having children, and longer working experience. These factors likely provide emotional support and stability, which enhance resilience [14]. This aligns with prior research suggesting that self-esteem and self-compassion are essential components of psychological resilience among nurses, particularly during high-stress events such as the COVID-19 pandemic [26]. Working in internal departments was associated with higher resilience, possibly due to the lower-stress nature of such environments compared to the intensive care and operating room units where critical decision-making demands increase work stress. This finding agrees with Xavier et al.'s study, which displayed that working in high-intensity units is responsible for higher levels of perceived stress, and thus lower resilience [27].

Limitations

While this study provides significant insights into the relationship between BI appreciation and psychological resilience among healthcare professionals, certain limitations should be acknowledged. The study uses a cross-sectional methodology, thus limiting the ability to establish causal relationships between BI appreciation, resilience, and other factors. Longitudinal studies are required to assess how these variables influence each other over time. Although the sample included both nurses and physicians, the generalizability of findings may be limited by its geographic or institutional context. A broader, more diverse sample across different healthcare systems and cultural contexts could yield more universally applicable findings. The reliance on self-reported measures for BI appreciation and resilience introduces potential biases, such as social desirability bias or inaccuracies in self-perception. Objective measures or mixed-method approaches could provide more robust data. Future research addressing these limitations would provide a more comprehensive understanding of these dynamics in healthcare settings.

## Conclusions

The results of this study show the existence of differences and similarities regarding nurses’ and physicians’ resilience. Nurses reported higher BI appreciation and resilience compared to physicians. However, in both groups, higher BI appreciation was one of the strongest positive predictors of resilience. The observed differences between nurses and physicians highlight the necessity for identifying ways to improve their BI appreciation, thus increasing the indirect association with both physical and psychological well-being. 

## Appendices

**Table 6:** Body Appreciation Scale-2 (BAS-2)

Body Appreciation Scale-2 (BAS-2) (Participants rated each item on a five-point Likert scale from 1 = Never to 5 = Always.)
I respect my body.
I feel good about my body.
I feel that my body has at least some good qualities.
I take a positive attitude toward my body.
I am attentive to my body’s needs.
I feel love for my body.
I appreciate the different and unique characteristics of my body.
My behavior reveals my positive attitude toward my body; for example, I hold my head high and smile.
I am comfortable in my body.
I feel like I am beautiful even if I am different from media images of attractive people (e.g., models, actresses/actors).

**Table 7:** Connor-Davidson Resilience Scale 10-item (CD-RISC-10)

Connor-Davidson Resilience Scale 10-item (CD-RISC-10) (Participants rated each item on a five-point Likert scale from 0 = Not true at all to 4 = True nearly all the time.)
I am able to adapt when changes occur.
I can deal with whatever comes my way.
I try to see the humorous side of things when I am faced with problems.
Having to cope with stress can make me stronger.
I tend to bounce back after illness, injury, or other hardships.
I believe I can achieve my goals, even if there are obstacles.
Under pressure, I stay focused and think clearly.
I am not easily discouraged by failure.
I think of myself as a strong person when dealing with life’s challenges and difficulties.
I am able to handle unpleasant or painful feelings like sadness, fear, and anger.
